# A Randomized Controlled Study of Robot-Assisted versus 3D Laparoscopic Radical Prostatectomy in Patients with Carcinoma Prostate

**DOI:** 10.1155/2023/4666116

**Published:** 2023-05-23

**Authors:** Ketan Kumar Kapoor, Anup Kumar

**Affiliations:** Department of Urology and Renal Transplant, Vardhman Mahavir Medical College and Safdarjung Hospital, New Delhi, India

## Abstract

**Materials and Methods:**

A prospective randomized comparative study was performed from 1st January 2020 to 30th June 2021. All patients included were diagnosed with localized/locally advanced ca prostate. 60 patients fulfilling the inclusion and exclusion criteria were randomized into 2 groups. Groups A and B included patients who underwent robot-assisted radical prostatectomy and 3D laparoscopic transperitoneal radical prostatectomy, respectively. Various demographic, intraoperative, postoperative, and follow-up parameters were collected. Outcomes were evaluated in the form of the trifecta (continence, potency, and BCR-free status) and pentafecta rates (trifecta with no perioperative complications and negative surgical margins) in between the two groups.

**Results:**

The mean operative time in Group A was 137.83 mins ± 17.27 compared to 148.20 mins ± 26.16 in Group B. Trifecta rates in Group A and Group B were 43.3%, 63.3%, and 76.6% and 40%, 53.3%, and 70% at 1, 3, and 6 months. Pentafecta rates in Group A and Group B were 36.6%, 53.3%, and 70% and 33.3%, 40%, and 53.3% at 1, 3, and 6 months. Complication rates were 10% in Group A and 13.3% in Group B, respectively. Only one patient in our study (Group B) had a positive surgical margin.

**Conclusions:**

We conclude from our comparative study, that both robot-assisted and 3D laparoscopic transperitoneal radical prostatectomy are feasible and efficacious treatment modalities for achieving acceptable trifecta and pentafecta rates in managing ca prostate with earlier continence and shorter urethrovesical anastomosis time in the robotic arm.

## 1. Introduction

Prostate cancer is the most common urological malignancy in the world. Treatment for prostate cancer depends upon several factors, such as whether the malignancy is localized, locally advanced, or metastatic [1]. Laparoscopic and robotic approaches have largely taken over open radical prostatectomy in recent years [2]. With the 3-dimensional laparoscopy technology, new stereoscopic vision enables better depth perception resulting in faster and safer outcomes, especially with intracorporeal suturing in the form of urethravesical anastomosis [3, 4]. However, laparoscopy has many limitations, and a steep learning curve is required for the surgeon. These shortcomings have led to the concept that robots may improve the precision and accuracy of anatomical dissection by offering enhanced freedom and easy maneuverability, thereby improving overall outcomes.

Only a few studies directly compare LRP (laparoscopic radical prostatectomy) and RARP (robot-assisted radical prostatectomy) from a single institution. This is the first Indian single-surgeon series reporting a prospective randomized comparison between 3D laparoscopic and robot-assisted radical prostatectomy for prostatic carcinoma.

## 2. Materials and Methods

**Figure 1:**
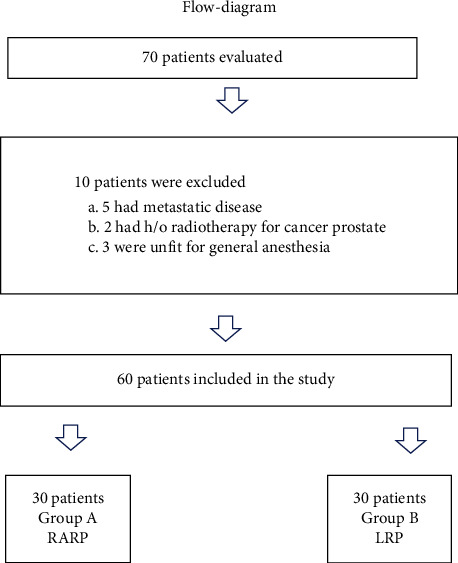
Flow diagram.

A total of 70 patients were evaluated in this prospective randomized comparative study, which was conducted at our institute from 1 January 2020 to 30 June 2021. 60 patients met the inclusion criteria. Patients were randomized into two groups using the computer-generated randomization table. Group A included patients who underwent robot-assisted radical prostatectomy and Group B included patients on whom laparoscopic transperitoneal radical prostatectomy was performed. Patients (age ≤76 years) with a life expectancy of a minimum of 10 years [1] having localized or locally advanced cancer prostate were included. Patients with metastatic, T4 disease, having received radiotherapy or hormonal therapy for prostate cancer and having any comorbidity precluding general anesthesia and laparoscopic surgery were excluded. Various demographic, intraoperative, postoperative, and follow-up parameters were collected. Ethical approval was obtained from the ethics committee (IEC/VMMC/SJH/Thesis/2020-03/CC-06) (Figure 1 flow diagram).

**Figure 2:**
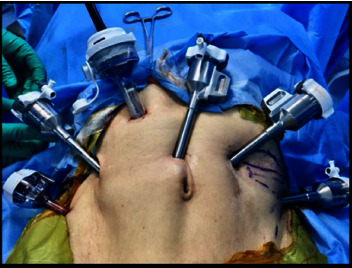
Port placement in RARP.

**Figure 3:**
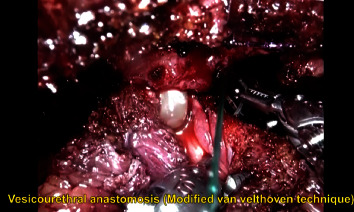
Urethro-vesical anastomosis (UVA).

**Figure 4:**
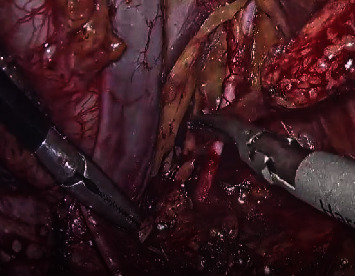
Extended pelvic lymph node dissection.

Surgeries were performed by a single urologist having extensive laparoscopic and robotic experience in both the groups. Both 3D (Storz HD) lap and RARP (four-arm da Vinci Xi Robotic System) were carried out using a transperitoneal posterior antegrade approach with the assistant port on the right side (Figure 2). The robotic approach required one extra assistant port. The urethrovesical anastomosis (UVA) was completed using continuous locking vicryl (V-Loc 3-0) (Figure 3). Extended pelvic lymph node dissection was performed in all cases (Figure 4). Pelvic drains were removed with a drain output of less than 30 ml/day. Patients were discharged with per urethral catheter in situ. Catheter removal was performed after 10 days.

Outcomes were evaluated by comparing the trifecta (continence, potency and BCR-free status) and pentafecta rates (trifecta along with no perioperative complications and negative surgical margins) amongst the two groups. Complications were graded using the modified Clavien−Dindo classification [5] with risk stratification via the D'Amico risk stratification system. Biochemical recurrence (BCR) was defined as two consecutive prostate-specific antigen (PSA) levels of >0.2 ng/ml. Functional outcomes were recorded at 1 month, 3 months, and 6 months after surgery. Continence was defined as the use of no pads in the past 1 month, while patients were able to achieve and maintain satisfactory erections for sexual intercourse in more than 50% of the attempts, with or without the use of PDE5 inhibitors, were considered potent. IIEF-5 questionnaire was used to compare the potency outcomes.

The SPSS-PC-25 version was used for data and statistical analysis. Quantitative data were expressed in mean ± standard deviation, while qualitative data was represented in percentages. Student's *t*-test (unpaired) or MannWhitney *U* test was used to test the normality distribution difference. The chi-square test or Fisher's exact test was used to test the statistical significance. *P*-value of less than 0.05 was considered statistically significant.

## 3. Results

A total of 60 patients were included and randomized into two groups. Demographic and perioperative comparisons are shown in Table 1. The mean age in Group A was 58, and in Group B was 60 (*p*=0.17). The mean body mass index (BMI), blood investigations, PSA values, and median lobes were comparable in both the groups. The mean prostate size on ultrasound in Group A was 52.0 cc, while in Group B, it was 42.90 cc (*p*=0.01) and the difference was significant. The mean Gleason's score on transrectal ultrasound (TRUS) biopsy in Group A was 6.97, and in Group B was 6.57 (*p*=0.04) and was significant. Of all patients, 26.7% had a GS of 6, 53.3% had a GS of 7 (3 + 4/4 + 3), 16.7% had a GS of 8 and 3.3% had a GS of 9 in Group A whereas 56.7% had a GS of 6, 30% had a GS of 7 (3 + 4/4 + 3), and 13.3% had a GS of 8 in Group B. The estimated blood loss in Group A was 160.67 ml, and in Group B was 154.5 ml. The *p* value was not significant. The mean urethrovesical anastomosis time was 16.80 ± 3.47 in Group A and in Group B, it was 19.83 ± 2.95 minutes (*p* value <0.01). 34 patients (20 in group A and 14 in group B) underwent neurovascular bundle preservation. Dissection was either interfascial or extrafascial on a case-to-case basis, with a focus on not compromising the oncological principles.

The complication rate in Group A was 10%; two patients (6.67%) had minor complications (grades 1 and 2), and one patient (3.3%) had grade 3a (major) complication (Table 2). In Group B, the complication rate was 13.3%; six patients (20%) had minor complications (grades 1 and 2) and one (3.3%) patient had a grade 3a (major) complication. Only one patient (Group B, 3.33%) had a positive surgical margin, for whom the adjuvant radiotherapy was planned (Table 3). The trifecta rates in Group A and Group B were 43.3%, 63.3%, and 76.6%, and 40%, 53.3%, and 70% at 1, 3, and 6 months. The pentafecta rates in Group A and Group B were 36.6%, 53.3%, and 70% and 33.3%, 40%, and 53.3% at 1, 3, and 6 months, respectively (Table 4).

## 4. Discussion

With its increasing availability, robotic surgery is now becoming the more preferred treatment modality for surgical management of localized/locally advanced carcinoma prostate. Given the significantly higher economic burden associated with the procedure, it is only imperative that the perioperative, oncological, and functional parameters of RARP, LRP, or open surgery are genuinely compared.

The current 3D laparoscopic systems are comparable to the robotic surgical systems in terms of good depth perception and reduction of surgeon stress. They are also more economical and easy to maintain. However, LRP is a technically demanding procedure requiring advanced laparoscopic dissection and suturing skills with a steeper learning curve.

Robot-assisted laparoscopy is now the go-to modality given the 3D vision, thereby increasing the six degrees of freedom, allowing better dexterity with instruments, tremor filters, and an ergonomic surgical console to reduce the surgeon's fatigue [2]. Although claims of superior functional and oncologic outcomes of RARP compared with other approaches are common in the current literature, almost all available data are derived from prospective nonrandomized or retrospective studies that provide low evidence [2, 6–9].

This is the first prospective randomized study comparing these two groups in terms of their outcomes. The preoperative and demographic data were comparable in both the groups and is similar to the previous studies. There was no significant difference in the mean operative time in the two groups; however, the urethrovesical anastomosis time was shorter in Group A. Alenizi et al. [10] reports the mean UVA time of 20 mins for RARP, which is longer in comparison to our study. Bove et al. [11] reports the mean operative time of 162 minutes and the mean UVA time of 24 minutes in the 3D laparoscopy arm, which is longer in comparison to the mean duration reported in Group B. Nerve sparing was performed in 20 (66.7%) patients in Group A, while in Group B, 14 (46.7%) patients underwent nerve-sparing surgery. The high anterior release of neurovascular bundles or veil of Aphrodite‖ technique of nerve-sparing, as described by Menon et al. [12] was used. Dissection was either interfascial or extrafascial on a case-to-case basis, with a focus on not compromising the oncological outcomes. The lymph node yield is an important factor which probably indicates an adequate dissection. We performed an extended lymph node dissection in all our cases. The average lymph node yield in Group A was 20.30 ± 3.39 (range 14–25), while in Group B, it was 18.40 ± 4.43(range 8–25). The yield in our study was comparable with that of the study by Porpiglia et al. [13], showing an average yield of 17 in both LRP and RARP arms.

The continence rates in Group A were 73%, 83%, and 96% and in Group B were 46.7%, 70%, and 90% at 1, 3, and 6 months, respectively. The *p* value for continence between the two groups was significant at 1 month. These results were comparable with the RCT published by Benelli et al. [14]. This may be due to the precise and meticulous dissection using robotic assistance around the neurovascular tissues with a shorter UVA time and a better preservation of the membranous urethral length.

BCR-free rates at 1, 3, and 6 months were 100% in all visits for both the groups, which were higher than those of other comparable studies, but a longer follow-up is suggested to establish significance.

Trifecta rates in Group A and Group B were 43.3%, 63.3%, and 76.6% and 40%, 53.3%, and 70% at 1, 3, and 6 months, respectively. Patel et al. [15] quoted 43.1%, 64.1%, 79.2%, and 83.1% trifecta rates at 6 weeks, 3, 6, and 12 months, respectively. Meanwhile, RCT by Porpiglia et al. [13] achieved rates of 40, 46, and 75% at 1, 3, and 6 months,respectively in the LRP arm of the study. These rates were comparable to our study.

Pentafecta rates in Group A and Group B were 36.6%, 53.3%, and 70% and 33.3%, 40%, and 53.3% at 1, 3, and 6 months, respectively. The 3 months pentafecta rates were comparable with Bove et al.'s [11] 3D LRP arm (49%), Patel et al.'s [15] RARP (51.8%), and Porpiglia et al.'s [13] LRP (45%); however, higher 6 months pentafecta rates were achieved in our study than those of other 3D LRP and RARP studies. The previously reported complication rates postrobotic radical prostatectomy have been estimated to be around 10% [16], which is similar to those in our study. In a reverse systematic review performed by Moretti et al. [17], minimally invasive surgery, especially RARP, shows better perioperative and complication results, which are associated with less complex cases, higher annual surgeon volume, and improved performance.

This study has limitations. The prospective nature of the study, the small sample size, and the short follow-up duration could affect the outcomes and alter decision-making. Multicenter randomized control trials with larger sample sizes and diverse study populations are recommended to validate our findings.

## 5. Conclusion

In this single-surgeon comparative study, both the robot-assisted and 3D laparoscopic transperitoneal radical prostatectomy are feasible and efficacious options in achieving acceptable pentafecta rates. RARP offers a significant edge in terms of the urethrovesical anastomosis time and the return to early continence with precise safety margins and comparable complications with the laparoscopic approach.

## Figures and Tables

**Table 1:** Demographic and perioperative outcomes.

Characteristics	RARP (*n* = 30) Group A	3D LRP (*n* = 30) Group B	*P* value
Mean age	58.03 ± 6.21	60.10 ± 5.49	0.17
BMI (kg/m^2^)	21.62 ± 2.71	21.39 ± 2.65	0.74
Hemoglobin (gm/dl)	12.73 ± 1.29	13.06 ± 1.51	0.35
S. creatinine (mg/dl)	0.95 ± 0.22	0.90 ± 0.28	0.24
PSA (ng/ml)	13.37 ± 6.15	11.87 ± 5.46	0.43
USG prostate size (cc)	52.07 ± 15.66	42.90 ± 16.89	0.01
Gleason's score (TRUS biopsy)	6.97 ± 0.76	6.57 ± 0.73	0.04
Mp-MRI			0.67
PIRADS IV	14 (53.8%)	11 (47.82%)	
PIRADS V	12 (46.1%)	12 (52.1%)	
D' Amico classification			0.24
Low risk	6 (20%)	12 (40%)	
Intermediate risk	16 (53.3%)	12 (40%)	
High risk	8 (26.7%)	6 (20%)	
IIEF-5	18.89 ± 4.19	19.03 ± 4.44	0.78
Operative time(minutes)	137.83 ± 1727	148.20 ± 26.16	0.11
UVA time (minutes)	16.80 ± 3.47	19.83 ± 2.95	<0.01
Blood loss (ml)	160.67 ± 113.25	154.5 ± 98.74	0.70
Nerve sparing			0.11
No	10 (33.3%)	16 (53.3%)	
Yes	20 (66.7%)	14 (46.7%)	
Drain removal day	2.47 ± 1.25	2.40 ± 1.10	0.97
Hospital stay (days)	3.50 ± 1.33	2.23 ± 0.57	0.90
Catheter removal days	11.50 ± 4.52	11.10 ± 3.6	0.89

**Table 2:** Comparison of complications between both the groups.

Type of complications	RARP (*n* = 30) Group A	3D LRP (*n* = 30) Group B	Clavien−Dindo grade
1 unit blood	1 (3.3%)	1 (3.3%)	2
Fever	0 (0%)	0 (0%)	1
High drain output	1 (3.3%)	1 (3.3%)	1
Ileus, 2 units of blood	0 (0%)	1 (3.3%)	2
Lower ureteric injury	1 (3.3%)	1 (3.3%)	3a
Pericatheter dye leak	1 (3.3%)	0 (0)	3a
Total	3 (10%)	4 (13.3%)	*P* value = 0.7

**Table 3:** Oncological outcomes.

Characteristics	RARP (*n* = 30) Group A	3D LRP (*n* = 30) Group B	*p* value
*T* Stage			0.5
pT2	28 (93.3%)	27 (90%)	
pT2b	1 (3.3%)	0	
pT3a	0	1 (3.3%)	
pT3b	1 (3.3%)	2 (6.7%)	
Lymph node yield	20.30 ± 3.39	18.40 ± 4.43	0.13
Gleason's score (final HPE)	6.90 ± 0.75	6.67 ± 0.71	0.22
Positive surgical margins	0	1 (3.3%)	1
Adjuvant therapy	0	1 (3.3%)	1

**Table 4:** Functional outcomes

Characteristics	Time	RARP (*n* = 30) Group A	3D LRP (*n* = 30) Group B	*p* value
Continence	1 month	22 (73.3%)	14 (46.7%)	0.03
	3 months	25 (83.3%)	21 (70%)	0.22
	6 months	28 (96.6%)	27 (90%)	1.0

Potency	1 month	13 (43.3%)	12 (40%)	0.79
	3 months	19 (63.3%)	16 (53.3%)	0.43
	6 months	24 (80%)	21 (70%)	0.55

BCR-free	1 month	30 (100%)	30 (100%)	—
	3 months	30 (100%)	30 (100%)	—
	6 months	30 (100%)	30 (100%)	—

Trifecta	1 month	13 (43.3%)	12 (40%)	0.79
	3 months	19 (63.3%)	16 (53.3%)	0.43
	6 months	23 (76.6%)	21 (70%)	0.55

Pentafecta	1 month	11 (36.67%)	10 (33.3%)	0.78
	3 months	16 (53.33%)	12 (40%)	0.30
	6 months	21 (70%)	16 (53.3%)	0.18

## Data Availability

The data used to support the findings of this study are available from the corresponding author upon request.

## Abbreviations

RARP: Robot-assisted radical prostatectomy
LRP: Laparoscopic radical prostatectomy
BCR: Biochemical recurrence
PSA: Prostate-specific antigen
BMI: Body mass index
TRUS: Transrectal ultrasound
IIEF: International Index of Erectile Function.
